# Dietary B vitamins and glioma: A case–control study based on Chinese population

**DOI:** 10.3389/fnut.2023.1122540

**Published:** 2023-03-02

**Authors:** Weichunbai Zhang, Jing Jiang, Xun Kang, Ce Wang, Feng Chen, Botao Zhang, Shenglan Li, Sijie Huang, Wenbin Li

**Affiliations:** Department of Neuro-Oncology, Cancer Center, Beijing Tiantan Hospital, Capital Medical University, Beijing, China

**Keywords:** B vitamins, glioma, case–control study, Chinese population, dose–response relationship

## Abstract

**Background:**

Dietary antioxidants have long been thought to be likely to prevent the development of gliomas. Previous studies have reported vitamin A, C, and E protective effects against gliomas. B vitamins, one of the main vitamins in the diet, are closely related to human health, but the association with gliomas has rarely been reported.

**Objective:**

This study aimed to evaluate the relationship between five B vitamins and glioma.

**Methods:**

In this Chinese population-based case–control study, 506 glioma cases and 506 matched (age and sex) controls were included. The dietary intake of study participants was assessed using a valid 111-item food frequency questionnaire. The intake of five B vitamins was calculated based on participants’ dietary information from the food frequency questionnaire. The logistic regression model was used to examine the association between B vitamins and glioma, and the restriction cubic spline evaluated the dose–response relationship between the two.

**Results:**

After adjusting for confounding factors, thiamine (OR = 0.09, 95%CI: 0.05–0.20), riboflavin (OR = 0.12, 95%CI: 0.06–0.25), nicotinic acid (OR = 0.24, 95%CI: 0.12–0.47), folate (OR = 0.07, 95%CI: 0.03–0.15) and biotin (OR = 0.14, 95%CI: 0.07–0.30) in the highest tertile were associated with a significantly decreased risk of glioma compared with the lowest tertile. The results of thiamine and biotin in glioma with different pathological types and grades were different. The restricted cubic spline function showed significant dose–response relationships between the intake of five B vitamins and the risk of glioma. When B vitamins exceeded a specific intake, the risk of glioma did not change.

**Conclusion:**

Our study suggests that higher dietary intake of thiamine, riboflavin, nicotinic acid, and folate are associated with a decreased risk of glioma, but the results of biotin are not consistent among different populations. In the future, prospective studies should be conducted better to validate the effects of B vitamins on gliomas.

## Introduction

B vitamins are a group of water-soluble micronutrients required by all forms of cellular life, from bacteria to humans (1). Unlike other nutrients, B vitamins are not classified based on chemical structural similarity but on their physiological functions in tissues and cells (2, 3). As cofactors of hundreds of enzymes, B vitamins are mainly involved in energy metabolism, DNA and protein synthesis, and signal molecule synthesis (3, 4). For B vitamins, the human body cannot synthesize itself, or the amount of synthesis is challenging to meet physiological needs, so it is still necessary to rely on various animal products and plants in the daily diet to obtain essential B vitamins (4). In addition, exercise, alcohol consumption, certain drugs, and changes in body status can also affect the need for B vitamins (4, 5). Thus, deficiencies in B vitamins remain a potential malnutrition problem worldwide (6).

Studies showed that the lack of B vitamins was closely related to cardiovascular diseases (7), neurodegenerative diseases (8, 9), kidney diseases (10), diabetes (11), and other chronic diseases. In recent years, the effects of B vitamins on cancer have also been discovered. Comin-Anduix et al. started subcutaneously injecting different doses of thiamine (vitamin B1) every day for 4 days after tumor implantation in mice. They found that low-dose thiamine can promote tumor growth, while high-dose thiamine can inhibit tumor growth. When thiamine supplementation was started on the 7th day before the tumor inoculation, the inhibitory effect was significantly enhanced, suggesting that thiamine has a preventive effect on cancer (12). A meta-analysis of 6,184 colorectal cancer cases also found that higher intakes of thiamine could significantly reduce the risk of colorectal cancer (Odds ratio (OR) = 0.76, 95%confidence interval (95%CI): 0.65–0.89) (13). Similar results were also found for riboflavin (vitamin B2). Zschabitz et al. found that total riboflavin intakes were negatively associated with the risk of colorectal cancer in a prospective cohort of 88,045 postmenopausal women recruited from 1993 to 1998 (Hazard ratio (HR) = 0.81, 95% CI: 0.66–0.99) (14). Lu et al. also found that riboflavin had a protective effect against gastric cancer (OR = 0.56, 95%CI: 0.39–0.81) in the case–control study based on the Korean population, especially in the female population (OR = 0.52, 95%CI: 0.28–0.97) (15). Although studies have also explored the relationship between nicotinic acid (vitamin B3) and digestive tract cancer, no significant results have been obtained (16). Chen et al. found that niacinamide, a nicotinic acid derivative, can reduce the incidence of non-melanoma skin cancer by 23% (17). In contrast, folate (vitamin B9) had a broader range of effects against cancer. Lin et al. conducted a meta-analysis by including 10 studies on folate intake and pancreatic cancer and found that increasing dietary folate intake by 100 μg/day was associated with a 7% decreased risk of pancreatic cancer (Relative risk (RR) = 0.93, 95%CI: 0.90–0.97) (18). Some studies have found that folate was closely related to lung cancer (19), endometrial cancer (20), and prostate cancer (21).

Although these studies suggested that B vitamins were closely related to cancer, few studies reported the relationship between B vitamins and glioma. The pathogenesis of glioma was still unclear. It was currently believed that this mechanism may be related to genetic mutation of genes (22, 23), disorder of cell signal pathway (24), and defects in DNA damage repair (25). Based on the available evidence, the physiological function of B vitamins also involved these aspects. Therefore, we could not ignore the impact of B vitamins on glioma. On the one hand, the general metabolic functions of B vitamins and their role in neurochemical synthesis may be considered to have specific effects on the brain (3), and the concentrations of B vitamins and their derivatives in the brain were significantly higher than in plasma (26–28). It seemed impossible to ignore the importance of B vitamins for the brain. On the other hand, the effects of other vitamins on glioma have been reported, especially vitamin A, vitamin C, and vitamin E. Epidemiological studies have shown that these vitamins with antioxidant effects have a certain preventive effect against glioma (29–31). In comparison, the evidence on B vitamins and glioma was minimal. Some studies have explored the association between B vitamins and brain tumors. Still, due to the variety of brain tumors, the results cannot represent the relationship between B vitamins and glioma, and these studies mainly focused on children (32–34). Therefore, we conducted a case–control study in a Chinese adult population to further explore the association between various B vitamins in the diet and glioma. This study evaluated the relationship between five B vitamins in the diet and glioma. It explored the dose–response relationship between the intake of B vitamins and the risk of glioma to provide the latest epidemiological evidence for vitamin prevention of glioma.

## Methods

### Study population

This case–control study was initiated in 2021 and completed in 2022 at the Beijing Tiantan Hospital, Affiliated with Capital Medical University. Based on previous studies, we assumed that about 80% of Chinese people took in B vitamins below the recommended level (35). We further assumed that adequate B vitamins would reduce the risk of glioma by 43% (31). With 80% power, and type I error of 0.05, the minimum sample size was calculated to be 256 cases and 256 healthy control subjects. Adult patients who were jointly diagnosed with glioma by neuro-oncology doctors and pathologists according to the 2021 neuro-oncology diagnostic criteria (36) about 3 months before the survey were included in the case group. On this basis, taking hormones and other drugs that interfere with diet, significant dietary behavior changes (such as weight loss, etc.), extreme energy intake (>5,000 or <400 kcal/day), pregnant women and nursing mothers, previous cancer (except glioma), and digestive, endocrine, and neurological conditions were excluded. The control group was recruited from the community’s healthy individuals who reported no glioma clinical manifestations and abnormalities in previous brain imaging studies. Each case was matched with the control by age (within 5 years) and sex during the study period. The corresponding controls were matched among the 506 eligible patients. In the end, a total of 506 pairs were included in the final statistical analysis. All participants provided informed consent, and the study protocol was approved by the Institutional Review Board of Beijing Tiantan Hospital, Capital Medical University (No. KY2022-203-02).

### Dietary assessment and calculation of B vitamins intake

The food frequency questionnaire was used to collect information on the type and amount of food intake of the research subjects in the past 12 months through face-to-face interviews. The food frequency questionnaire has been validated in previous studies, and its authenticity and reliability can meet the purpose of the study (37). Based on this, referring to the existing articles on diet and glioma, we added and deleted several foods to make them more suitable for the study. The food frequency questionnaire in this study included refined grains (*n* = 9), whole grains (*n* = 2), tubers (*n* = 2), legumes and products (*n* = 5), vegetables (*n* = 23), fungi and algae (*n* = 4), fruits (*n* = 20), red meat (*n* = 4), poultry (*n* = 2), animal offal (*n* = 4), fish and seafood (*n* = 5), egg (*n* = 1), dairy products (*n* = 4), nut (*n* = 4), sweet food (*n* = 5), sugary drink (*n* = 3), tea and coffee (*n* = 3), condiment (*n* = 4), curing food (*n* = 3), processed products (*n* = 3), and alcohol (*n* = 4), a total of 114 items, basically covering the daily type of diet. The intake assessment for each food item consisted of three aspects: whether or not, the frequency of intake (daily/weekly/monthly), and single intake. In the questionnaire, g or ml was used as a unit to measure food intake, and pictures of different food volumes and qualities were provided as references to help subjects accurately assess intake. The daily intake of each food was calculated according to the frequency and single intake.

Five B vitamins were involved in the study, including thiamine, riboflavin, nicotinic acid, folate, and biotin (vitamin B7). The intakes of the five B vitamins were calculated using the China Food Composition Tables (38). Chinese Food Composition Tables provided data on B vitamins and energy in each food. Combined with the daily intake of each food item, the total daily intake of five B vitamins and energy can be calculated.

### Other variables

In addition to the food frequency questionnaire, all participants were asked to complete other surveys, including basic information, disease history, and lifestyle habits. Basic information mainly included date of birth, sex, education levels (primary school and below, secondary school and university and above), occupation (manual workers, mental workers, or others), and household income (below 3,000 ¥/month, 3,000–10,000 ¥/month, or above 10,000 ¥/month). Disease history included allergies, head trauma, and family cancer, which have been reported as “yes” or “no.” Lifestyle habits included smoking status and physical activity. The subjects were classified as never smoking, former smoking, or current smoking according to their current smoking status. Physical activity was assessed using the International Physical Activity Questionnaire (IPAQ) (39), and metabolic equivalents were calculated to classify physical activity into low, moderate, or violent. In addition, according to previous studies, the proximity of residence to electromagnetic fields and or broadcast antennas may also be a risk factor for glioma, so defining this condition as living in a high-risk area was also listed as one of the potential confounding factors. For physical measurement, while the subjects were being examined, researchers measured their weight and height using calibrated instruments to calculate body mass index (BMI). BMI was measured by weight (kg)/height squared (m^2^).

To ensure survey quality, all surveys were conducted by one-on-one interviews with uniformly trained investigators with medical or epidemiological education.

### Statistical analysis

We used the *t*-test for continuous variables and the *χ*^2^ test for categorical variables to compare the general characteristics of the case and control groups. The Spearman correlation coefficient was used to evaluate the correlation between five B vitamins. We divided them into three groups based on the distribution of B vitamin intake. To explore the association between B vitamins and glioma, we used the lowest group as a reference and calculated the OR and 95%CI of each group by logistic regression. The univariate model (Model 1) was a crude model without adjusting for any confounding factors. The multivariate model (Model 2) adjusted for factors associated with the risk of glioma and B vitamin intake: age, BMI, occupation, education level, household income, high-risk residential areas, smoking status, history of allergies, history of head trauma, family history of cancer, physical activity, and energy intake.

We conducted a series of sensitivity analysis to test the robustness of our estimates by excluding participants with different ages, different sexes, different BMI, middle school and below, below 3,000 ¥/month, smoking, history of allergy or family history of cancer, and repeated regression analysis. In addition, to overcome the inherent limitations of B vitamins analysis as grade variables, the restricted cubic spline function was used to model the dose–response relationship in the multivariate adjustment model, with four nodes located at the 20th, 40th, 60th, and 80th percentiles of B-vitamin intake. The 10th percentile was used as the reference group (OR = 1) (40).

All analyses were performed using SPSS 26.0 and R 4.1.1. All reported *p*-values were 2-sided, and the significance level was set at *p* < 0.05.

## Results

### Characteristics of the study population and B vitamins

A total of 506 glioma patients were included in this study, including 7 patients with grade I, 98 cases with grade II, 73 cases with grade III, 255 cases with grade IV, and 73 cases that could not judge the pathological grade. The glioma population of each pathological grade and the corresponding control group had similar age distribution, and the sex composition was utterly consistent. Overall, glioma patients had higher BMI (*p* < 0.001), slightly fewer education levels (*p* < 0.001), more smoking (*p* = 0.039), more physical activity (*p* < 0.001), and were less likely to have allergies (*p* < 0.001) but more likely to have cancer in their families (*p* = 0.001). There were also differences in occupation (*p* = 0.024) and household income (*p* < 0.001; Table 1).

**Table 1:** Basic characteristics of the study participants.

	Grade I + II			Grade III			Grade IV			Others			Value of *p*^a,b^
	Case	Control	Value of *p*^a^	Case	Control	Value of *p*^a^	Case	Control	Value of *p*^a^	Case	Control	Value of *p*^a^	
Age (years)	38.50 ± 12.76	37.14 ± 12.73	0.443	41.40 ± 11.32	39.71 ± 11.09	0.365	44.38 ± 13.29	42.86 ± 13.02	0.193	43.62 ± 13.32	42.36 ± 12.86	0.562	0.072
Sex, (%)			1.000			1.000			1.000			1.000	1.000
Male	61.9	61.9		58.9	58.9		56.1	56.1		45.2	45.2		
Female	38.1	38.1		41.1	41.1		43.9	43.9		54.8	54.8		
BMI (kg/m^2^)	24.49 ± 3.06	23.14 ± 3.62	0.004	23.80 ± 3.05	23.05 ± 3.19	0.144	24.00 ± 3.35	23.12 ± 3.16	0.002	23.70 ± 3.35	22.69 ± 3.27	0.067	<0.001
High-risk residential area, (%)			0.490			0.674			0.911			0.575	0.534
Yes	21.9	18.1		20.5	17.8		19.2	19.6		28.8	24.7		
No	78.1	81.9		79.5	82.2		80.8	80.4		71.2	75.3		
Occupation, (%)			0.190			0.597			0.095			0.009	0.024
Manual workers	29.5	21.9		27.4	23.3		22.0	20.4		37.0	15.1		
Mental workers	60.0	60.0		53.4	61.6		52.5	61.2		39.7	57.5		
Others	10.5	18.1		19.2	15.1		25.5	18.4		23.3	27.4		
Education level, (%)			0.009			0.005			<0.001			0.001	<0.001
Primary school and below	3.8	4.8		8.2	1.4		5.9	2.4		13.7	1.4		
Middle school	42.9	22.9		41.1	23.3		40.0	25.1		45.2	30.1		
University and above	53.3	72.4		50.7	75.3		54.1	72.5		41.1	68.5		
Household income, (%)			<0.001			<0.001			<0.001			0.396	<0.001
<3,000 ¥/month	11.4	17.1		11.0	21.9		6.7	18.4		16.4	15.1		
3,000–10,000 ¥/month	76.2	49.5		78.0	46.6		78.4	47.8		64.4	56.1		
>10,000 ¥/month	12.4	33.3		11.0	31.5		14.9	33.7		19.2	28.8		
Smoking status, (%)			0.197			0.151			0.239			0.229	0.039
Never smoking	63.8	75.2		74.0	71.2		71.8	74.9		68.5	80.8		
Former smoking	13.3	9.6		12.3	5.5		13.7	9.0		9.6	5.5		
Current smoking	22.9	15.2		13.7	23.3		14.5	16.1		21.9	13.7		
History of allergies, (%)			0.092			0.149			0.116			0.007	<0.001
Yes	5.7	12.4		9.6	17.8		8.6	12.9		5.5	20.5		
No	94.3	87.6		90.4	82.2		91.4	87.1		94.5	79.5		
History of head trauma, (%)			0.083			0.796			0.310			0.042	0.474
Yes	15.2	7.6		11.0	12.3		9.0	11.8		13.7	4.1		
No	84.8	92.4		89.0	87.7		91.0	88.2		86.3	95.9		
Family history of cancer, (%)			0.862			0.003			0.022			0.341	0.001
Yes	20.0	19.0		39.7	17.8		31.8	22.7		28.8	21.9		
No	80.0	81.0		60.3	82.2		68.2	77.3		71.2	78.1		
Physical Activity, (%)			<0.001			<0.001			<0.001			<0.001	<0.001
Low	16.2	41.9		12.3	39.7		14.1	47.4		9.6	52.1		
Moderate	41.9	37.1		42.5	42.5		42.0	35.7		37.0	31.5		
Violent	41.9	21.0		45.2	17.8		43.9	16.9		53.4	16.4		

^a^*p*-values were derived from Student’s *t*-tests for continuous variables according to the data distribution and the chi-square test for the classified variables.

b Results of the overall case group and the overall control group.

The education level (*p* < 0.05), household income (*p* < 0.001), and physical activity (*p* < 0.001) of glioma patients with different pathological grades were the same as the general population. In addition, compared with the corresponding control group, the group with grade I + II glioma had a higher BMI (*p* = 0.004), the group with grade III glioma had a higher percentage of family history of cancer (*p* = 0.003), the group with grade IV glioma had a higher BMI (*p* = 0.002) and a higher percentage of family history of cancer (*p* = 0.022), and the group with other glioma had more manual workers (*p* = 0.009), a lower history of allergies (*p* = 0.007) and a higher history of head trauma (*p* = 0.042). There were no significant differences in others (Table 1).

**Figure 1:**
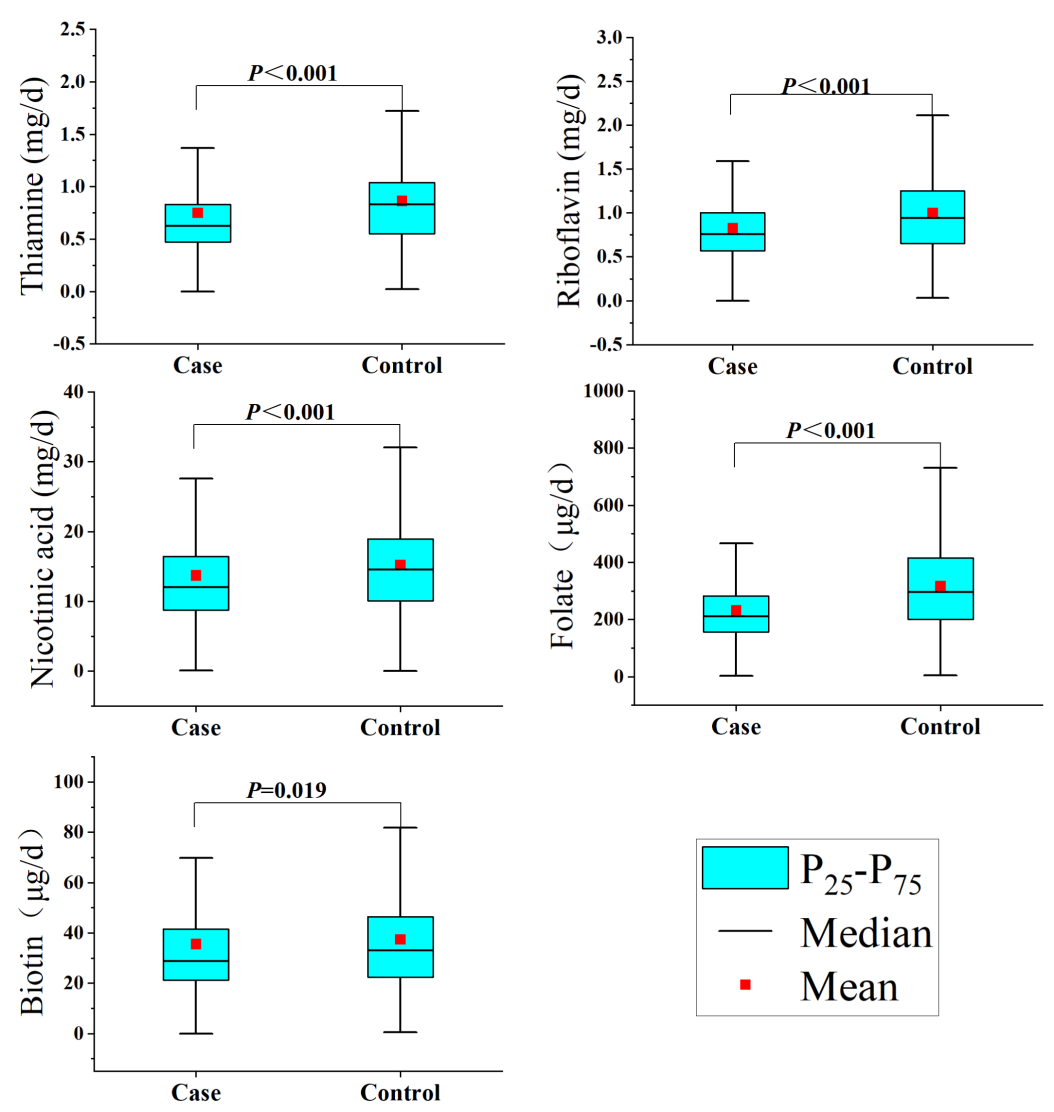
B vitamins intake among study participants.

Regarding the intake of B vitamins, as shown in Table 2, the intakes of thiamine, riboflavin, nicotinic acid, folate, and biotin in the control group were significantly higher than those in the case group (Figure 1). In addition, there was a significant correlation between the intake of the individual B vitamins (Spearman coefficients ranged from 0.559 to 0.798; Supplementary Table S1).

**Table 2:** Adjusted ORs and 95% CIs for the association between B vitamins and glioma.

B vitamins	T1	T2	T3	Continuous^c^	*P_-trend_*
Thiamine (mg/day)	≤0.57	0.57–0.88	>0.88		
Case/Control	206/132	186/151	114/223		
Model 1^a^	1	0.79 (0.57–1.08)	0.34 (0.25–0.47)	0.95 (0.92–0.98)	<0.001
Model 2^b^	1	0.38 (0.21–0.68)	0.09 (0.05–0.20)	0.91 (0.86–0.96)	<0.001
Riboflavin (mg/day)	≤0.68	0.68–0.99	>0.99		
Case/Control	201/137	177/160	128/209		
Model 1^a^	1	0.72 (0.52–0.99)	0.38 (0.28–0.54)	0.92 (0.89–0.95)	<0.001
Model 2^b^	1	0.48 (0.26–0.87)	0.12 (0.06–0.25)	0.70 (0.64–0.78)	<0.001
Nicotinic acid (mg/day)	≤10.68	10.68–15.88	>15.88		
Case/Control	195/143	169/170	142/193		
Model 1^a^	1	0.74 (0.54–1.01)	0.55 (0.40–0.74)	0.87 (0.80–0.95)	<0.001
Model 2^b^	1	0.40 (0.23–0.69)	0.24 (0.12–0.47)	0.62 (0.50–0.77)	<0.001
Folate (μg/day)	≤195.82	195.82–307.57	>307.57		
Case/Control	218/120	193/144	95/242		
Model 1^a^	1	0.71 (0.50–1.00)	0.22 (0.15–0.31)	0.64 (0.57–0.71)	<0.001
Model 2^b^	1	0.57 (0.31–1.07)	0.07 (0.03–0.15)	0.31 (0.23–0.42)	<0.001
Biotin (μg/day)	≤24.62	24.62–39.05	>39.05		
Case/Control	180/158	179/158	147/190		
Model 1^a^	1	0.98 (0.73–1.33)	0.67 (0.49–0.92)	0.97 (0.92–1.02)	0.007
Model 2^b^	1	0.48 (0.27–0.86)	0.14 (0.07–0.30)	0.76 (0.66–0.88)	<0.001

a Model 1: Unadjusted model.

b Model 2: Adjusted for age, BMI, occupation, education level, household income, high-risk residential areas, smoking status, history of allergies, history of head trauma, family history of cancer, physical activity, and energy intake.

c Thiamine, riboflavin per 0.1 mg/day increments, nicotinic acid per 5 mg/day increment, folate per 100 μg/day increments, biotin per 10 μg/day increments.

### Association between B vitamins and glioma

The association results between the five B vitamins and glioma are shown in Table 2. In model 1, the intake of each B vitamin was significantly associated with the risk of glioma. After adjusting for age, BMI, and other variables (Model 2), the results for the categorical variable of B vitamin intake showed that, compared to the first tertile, the third tertile of thiamine was associated with a decreased risk of glioma (OR = 0.09, 95%CI: 0.05–0.20), the third tertile of riboflavin was associated with a decreased risk of glioma (OR = 0.12, 95%CI: 0.06–0.25), the third tertile of nicotinic acid was associated with a decreased risk of glioma (OR = 0.24, 95%CI: 0.12–0.47), the third tertile of folate was associated with a decreased risk of glioma (OR = 0.07, 95%CI: 0.03–0.15), and the third tertile of biotin was associated with a decreased risk of glioma (OR = 0.14, 95%CI: 0.07–0.30). The results of the analysis of the continuous variables showed that for each 0.1 mg/day increase in thiamine, the risk of glioma decreased by 9% (OR = 0.91, 95%CI: 0.86–0.96), and for each 0.1 mg/day increase in riboflavin, the risk of glioma decreased by 30% (OR = 0.70, 95%CI: 0.64–0.78), and for each 5 mg/day increase in nicotinic acid, the risk of glioma decreased by 38% (OR = 0.62, 95%CI: 0.50–0.77), and for each 100 μg/day increase in folate, the risk of glioma decreased by 69% (OR = 0.31, 95%CI: 0.23–0.42), and for each 10 μg/day increase in biotin, the risk of glioma decreased by 24% (OR = 0.76, 95%CI: 0.66–0.88; Table 2).

### B vitamins and pathological classification and grading of glioma

Analysis of pathological subtypes of glioma showed that for astrocytoma, riboflavin (OR = 0.47, 95%CI: 0.28–0.78), nicotinic acid (OR = 0.56, 95%CI: 0.33–0.96), folate (OR = 0.11, 95%CI: 0.03–0.43) and biotin (OR = 0.55, 95%CI: 0.36–0.84) were associated with a significantly decreased risk, but the result of thiamine was not significant (OR = 0.91, 95% CI: 0.81–1.01). For glioblastoma, thiamine (OR = 0.89, 95%CI: 0.79–0.99), riboflavin (OR = 0.79, 95%CI: 0.68–0.92), nicotinic acid (OR = 0.55, 95%CI: 0.36–0.83), and folate (OR = 0.24, 95%CI: 0.13–0.47) were associated with a significantly decreased risk, but the result of biotin was not significant (OR = 0.81, 95% CI: 0.65–1.01). Due to the small sample size of oligodendroglioma, further analysis was not possible (Table 3).

**Table 3:** Adjusted ORs and 95% CIs for the association between B vitamins and glioma of different pathological classifications.

Pathological classification^c^	Model 1^a^	Value of *p*	Model 2^b^	Value of *p*
Astrocytoma				
Thiamine	0.97 (0.93–1.02)	0.274	0.91 (0.81–1.01)	0.084
Riboflavin	0.91 (0.86–0.97)	0.004	0.47 (0.28–0.78)	0.004
Nicotinic acid	0.92 (0.78–1.08)	0.297	0.56 (0.33–0.96)	0.035
Folate	0.58 (0.44–0.76)	<0.001	0.11 (0.03–0.43)	0.001
Biotin	0.93 (0.83–1.04)	0.212	0.55 (0.36–0.84)	0.006
Glioblastoma				
Thiamine	0.92 (0.88–0.97)	0.001	0.89 (0.79–0.99)	0.044
Riboflavin	0.93 (0.89–0.97)	<0.001	0.79 (0.68–0.92)	0.002
Nicotinic acid	0.84 (0.73–0.96)	0.011	0.55 (0.36–0.83)	0.005
Folate	0.62 (0.53–0.74)	<0.001	0.24 (0.13–0.47)	<0.001
Biotin	0.95 (0.87–1.03)	0.218	0.81 (0.65–1.01)	0.065

a Model 1: Unadjusted model.

b Model 2: Adjusted for age, BMI, occupation, education level, household income, high-risk residential areas, smoking status, history of allergies, history of head trauma, family history of cancer, physical activity, and energy intake.

c Thiamine, riboflavin per 0.1 mg/day increments, nicotinic acid per 5 mg/day increment, folate per 100 μg/day increments, biotin per 10 μg/day increments.

The association between B vitamins and the pathological grade of glioma showed similar results. For low-grade gliomas, riboflavin (OR = 0.54, 95%CI: 0.37–0.79), nicotinic acid (OR = 0.50, 95%CI: 0.29–0.89), and folate (OR = 0.16, 95%CI: 0.06–0.44) were associated with a decreased risk, but the results of thiamine (OR = 0.90, 95% CI: 0.80–1.01) and biotin (OR = 0.75, 95% CI: 0.51–1.11) were not significant. For high-grade gliomas, thiamine (OR = 0.88, 95%CI: 0.82–0.96), riboflavin (OR = 0.73, 95%CI: 0.64–0.84), nicotinic acid (OR = 0.63, 95%CI: 0.46–0.86), folate (OR = 0.26, 95%CI: 0.16–0.43) and biotin (OR = 0.79, 95%CI: 0.65–0.96) were associated with a decreased risk (Table 4).

**Table 4:** Adjusted ORs and 95% CIs for the association between B vitamins and glioma of different grades.

Glioma grading^c^	Model 1^a^	Value of *p*	Model 2^b^	Value of *p*
Low grade				
Thiamine	0.96 (0.91–1.02)	0.231	0.90 (0.80–1.01)	0.055
Riboflavin	0.89 (0.82–0.96)	0.003	0.54 (0.37–0.79)	0.001
Nicotinic acid	0.85 (0.69–1.04)	0.113	0.50 (0.29–0.89)	0.018
Folate	0.60 (0.46–0.78)	<0.001	0.16 (0.06–0.44)	<0.001
Biotin	0.97 (0.87–1.08)	0.572	0.75 (0.51–1.11)	0.151
High grade				
Thiamine	0.95 (0.91–0.98)	0.002	0.88 (0.82–0.96)	0.003
Riboflavin	0.94 (0.90–0.97)	<0.001	0.73 (0.64–0.84)	<0.001
Nicotinic acid	0.89 (0.80–0.99)	0.026	0.63 (0.46–0.86)	0.003
Folate	0.65 (0.57–0.74)	<0.001	0.26 (0.16–0.43)	<0.001
Biotin	0.97 (0.91–1.04)	0.407	0.79 (0.65–0.96)	0.018

a Model 1: Unadjusted model.

b Model 2: Adjusted for age, BMI, occupation, education level, household income, high-risk residential areas, smoking status, history of allergies, history of head trauma, family history of cancer, physical activity, and energy intake.

c Thiamine, riboflavin per 0.1 mg/day increments, nicotinic acid per 5 mg/day increment, folate per 100 μg/day increments, biotin per 10 μg/day increments.

### Sensitivity analysis

The results of sensitivity analysis showed that after excluding participants with different ages, different sexes, different BMI, middle school and below, below 3,000 ¥/month, smoking, history of allergy or family history of cancer, we observed that most results of thiamine, riboflavin, nicotinic acid and folate were consistent with the overall results. However, biotin was not significant in people with low BMI (Supplementary Table S2).

### Dose–response relationship

**Figure 2:**
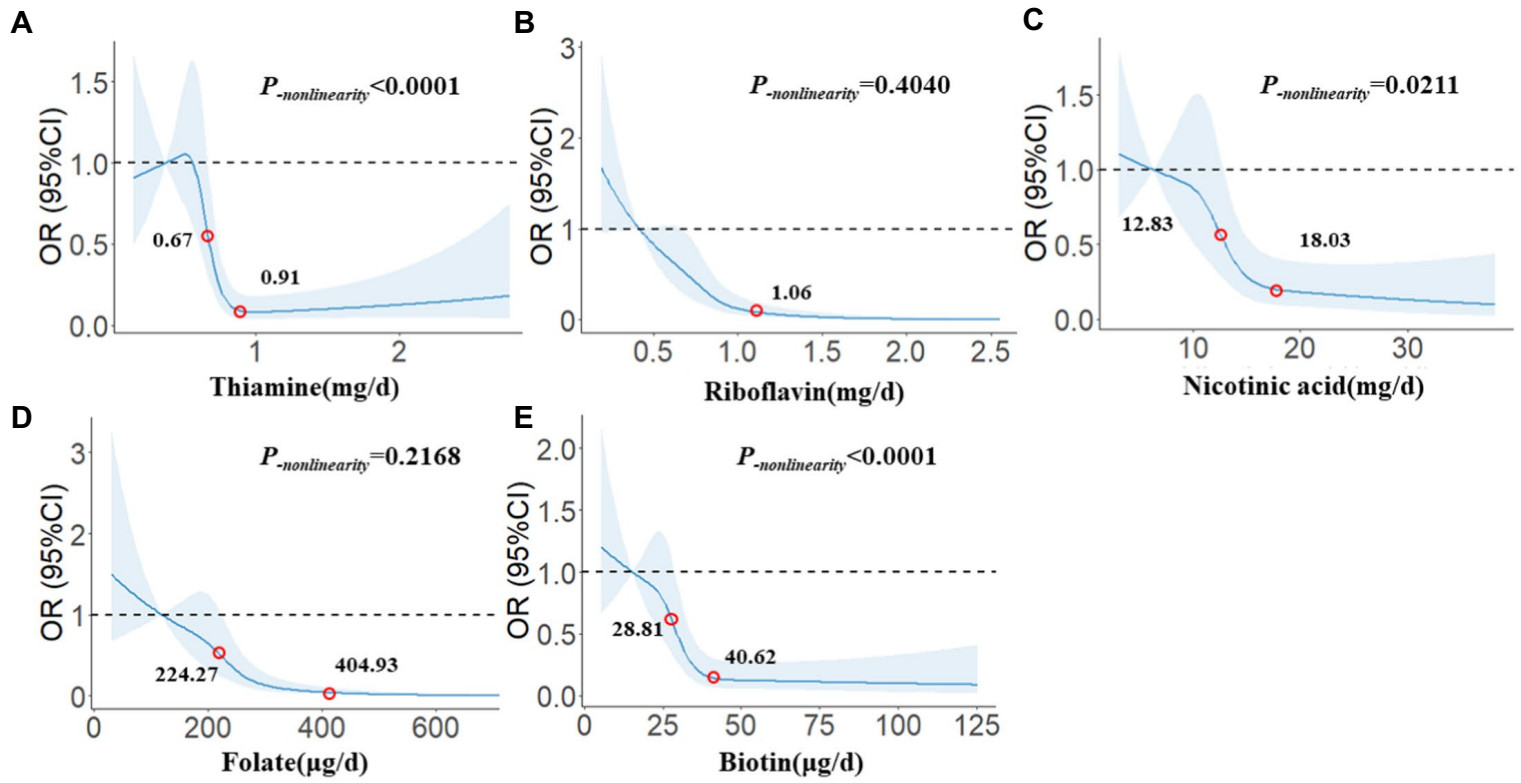
The restricted cubic spline for the associations between B vitamins and glioma. The lines represent adjusted odds ratios based on restricted cubic splines for the intake in the regression model. Knots were placed at the 20th, 40th, 60th, and 80th percentiles of the B vitamins intake, and the reference value was set at the 10th percentile. The adjusted factors were the same as in Model 2. **(A)** Thiamine, **(B)** Riboflavin, **(C)** Nicotinic acid, **(D)** Folate, **(E)** Biotin.

In Figure 2, we used restricted cubic splines to describe the relationships between B vitamins and glioma. There were significant dose–response relationships between the intake of five B vitamins and the risk of glioma. There was a nonlinear dose–response relationship between thiamine and the risk of glioma, and when intake exceeded 0.67 mg/day, the risk of glioma decreased significantly with increasing intake. After the intake exceeded 0.91 mg/day, the risk of glioma tended to stabilize (*P_-nonlinearity_* < 0.0001). There was a linear dose–response relationship between riboflavin and the risk of glioma, and the risk of glioma decreased significantly with the increase in intake. After the intake exceeded 1.06 mg/day, the risk of glioma tended to stabilize (*P_-nonlinearity_* = 0.4040). There was a nonlinear dose–response relationship between nicotinic acid and the risk of glioma, and when intake exceeded 12.83 mg/day, the risk of glioma decreased significantly with increasing intake. After the intake exceeded 18.03 mg/day, the risk of glioma tended to stabilize (*P_-nonlinearity_* = 0.0211). There was a linear dose–response relationship between folate and the risk of glioma. When the intake exceeded 224.27 μg/day, the risk of glioma decreased significantly with the increase in intake. After the intake exceeded 404.93 μg/day, the risk of glioma tended to stabilize (*P_-nonlinearity_* = 0.2168). There was a nonlinear dose–response relationship between biotin and the risk of glioma, and when intake exceeded 28.81 μg/day, the risk of glioma decreased significantly with increasing intake. After the intake exceeded 40.62 μg/day, the risk of glioma tended to stabilize (*P_-nonlinearity_* < 0.0001).

## Discussion

Previous observational studies on vitamins and glioma focused on vitamins A, C, and E, while studies on B vitamins were relatively rare. Our study evaluated the association between the intake of five B vitamins and glioma in the Chinese population. The results showed that thiamine, riboflavin, nicotinic acid, folate, and biotin were significantly negatively associated with the risk of glioma. The results of different pathological classifications were different. Thiamine only had a significant impact on glioblastoma. Similarly, it also existed between biotin and astrocytoma, and riboflavin and folate seemed to have a greater effect against astrocytoma. The results of different pathological grades were similar to those of pathological types. Thiamine and biotin only had significant effects against high-grade gliomas and had no significant correlation with low-grade gliomas, but the effects of riboflavin, nicotinic acid, and folate had a greater effect against low-grade glioma. The results of sensitivity analysis suggested that thiamine, riboflavin, nicotinic acid, and folate had relatively robust associations with glioma. Still, the results of biotin were not consistent with those of the general population, suggesting that its effects on glioma were different. The restricted cubic spline model further confirmed significant dose–response relationships between the five B vitamins and the risk of glioma, in which the dose–response relationships between thiamine, nicotinic acid, biotin, and glioma were nonlinear. In contrast, the dose–response relationships between riboflavin, folate, and glioma were linear.

Based on this, epidemiological studies have mainly focused on thiamine and nervous system diseases. Jung et al. found that thiamine deficiency can lead to Wernicke’s encephalopathy, which was often manifested as headache, inattention, irritability, confusion, apathy, impaired consciousness of the immediate situation, coma, and other obvious neurological symptoms (41). But most studies on thiamine and cancer have focused on the body. Liu et al. based on a meta-analysis of seven articles and 6,184 colorectal cancer patients, found that high-dose thiamine intake significantly reduced the risk of colorectal cancer (OR = 0.76, 95%CI: 0.65–0.89) (13). Cancarini et al. found that high intake of thiamine was associated with a lower risk of breast cancer (RR = 0.61, 95%CI: 0.38–0.97) in a prospective cohort of 10,786 women followed for an average of 16.5 years, especially in estrogen receptor-negative and progesterone receptor-negative breast cancer (42). However, there have been few reported studies on thiamine and glioma. Our study might be the first to find that higher dietary thiamine intake can significantly reduce the risk of glioma (OR = 0.09, 95%CI: 0.05–0.20), but this effect varied among different pathological types and grades of gliomas. It was protective against high-grade gliomas, such as glioblastoma, but similar results were not found in astrocytomas or other low-grade gliomas. The results of the dose–response relationship suggested that the relationship between thiamine intake and the risk of glioma was non-linear, with no change in the risk of glioma beyond 0.91 mg/day (*P_-nonlinearity_* < 0.0001). But the mechanism of the effect of thiamine on glioma was still unclear, which was related to the antioxidation of thiamine *in vivo* (43, 44). Oxidative stress and the production of free radicals may promote the occurrence and development of cancer (45), and gliomas were no exception (46). Cell experiments showed that thiamine significantly decreased the malondialdehyde level and increased the levels of superoxide dismutase and catalase in C6 rat glioma cells, inhibiting oxidative stress to some extent (47).

Compared with thiamine, riboflavin was more closely related to cancer, especially in digestive tract cancer, riboflavin showed a certain preventive effect. In a case–control study involving 756 controls and 377 cases of gastric cancer, Lu et al. assessed dietary riboflavin intake through a food frequency questionnaire. They found that riboflavin was significantly negatively correlated with the risk of glioma (OR = 0.56, 95%CI: 0.39–0.81) and interacted with methionine synthetase reductase (15). Based on the Women’s Health Initiative Observational Study cohort, Zschabitz et al. conducted a colorectal cancer study. They found that higher riboflavin intake significantly reduced the risk of colorectal cancer (RR = 0.81, 95% CI: 0.66–0.99), but this association was statistically significant only when the intake exceeded 3.97 mg/day (14). Similar results were also verified in a meta-analysis that included 8 articles (48). However, the effect of riboflavin on cancer varied by location, and its protective effect has not been consistent in studies on other cancer sites (49, 49). There were still few reports on riboflavin and glioma, and only studies have been conducted in the Middle East. Heydari et al. found no significant association between riboflavin and glioma by comparing riboflavin intake in 128 gliomas and 256 healthy individuals in a hospital case–control study in Iran (OR = 0.57, 95%CI: 0.18–1.78) (31). This was different from our results. In this study, there was a significant negative correlation between riboflavin and the risk of glioma (OR = 0.12, 95%CI: 0.06–0.25), and consistent results were obtained in different pathological subtypes and grades of glioma. This was not contradictory to the Iranian study. On the one hand, there were dietary differences between the two regions, and dietary riboflavin intake was significantly different. There was no difference in riboflavin intake in the Iranian population between glioma patients and healthy people (case: 2.50 ± 0.58 mg/day, control: 2.60 ± 1.29 mg/day), and was much higher than that in our study population (51). On the other hand, the dose–response relationship showed that when riboflavin intake exceeds 1.06 mg/day, the risk of glioma no longer changed, so it was necessary to repeat this study in different populations. Riboflavin can participate in the glutathione redox cycle, maintain the oxidative/antioxidant balance, resist oxidative stress, and inhibit cancer development (52). Riboflavin also had a synergistic effect with folate in DNA synthesis and repair (53, 54), which provided a possible explanation for riboflavin to prevent the occurrence and development of glioma.

Based on the beneficial effects of nicotinic acid on the skin, most cancer studies related to nicotinic acid have also focused on areas rich in epithelial tissue. Several clinical trials and animal studies have shown that nicotinic acid can prevent skin cancer, especially non-melanoma skin cancer, by promoting DNA repair, inhibiting pro-inflammatory mediators, and reducing light damage to the skin (43, 55, 56). Oral nicotinamide was found to be safe and effective in reducing the incidence of new non-melanoma skin cancer in high-risk patients in phase 3, double-blind, randomized, controlled trials (17). In addition to skin cancer, nicotinic acid had a similar effect in other epithelial-rich areas, such as esophageal cancer (57) and endometrial cancer (58). We also found the preventive effect of nicotinic acid in glioma populations. Higher nicotinic acid intake reduced the risk of glioma by 76% (OR = 0.24, 95%CI: 0.12–0.47) and had roughly the same effect on astrocytoma and glioblastoma. The dose–response relationship suggested that the effect of nicotinic acid against the risk of glioma was slightly different before and after the intake of 18.03 mg/day. At present, the mechanism of nicotinic acid inhibiting the occurrence and development of glioma was not clear. Yang et al. observed that nicotinic acid selectively targeted glioblastoma cells and retained most normal glial cells and neurons through overnight treatment of U251 glioblastoma cells with different concentrations of nicotinic acid. It was also observed that nicotinic acid decomposes F-actin stress fibers, which in turn affects cell-matrix adhesion, suggesting that nicotinic acid can inhibit the invasion of glioma cells (59). Li et al. found that nicotinic acid can cause the loss of mesenchymal phenotype in U251 glioblastoma cells. It has been found to inhibit glioma invasion *in vitro* and *in vivo*. The primary mechanism was that nicotinic acid promoted snail1 degradation and enhanced intercellular adhesion, indicating that epithelial-mesenchymal transition in glioma cells was inhibited (60).

Of all the B vitamins, folate had the most extensive effect on cancer. Increasing folate intake within the physiological dose had a protective effect against cancer (61). Epidemiological studies have shown that higher folate intake can significantly reduce the risk of colorectal cancer (62), pancreatic cancer (18), and endometrial cancer (20). But the reverse results have been obtained in the study of lung cancer (19) and prostate cancer (21). Compared with other B vitamins, there were more studies on folate and brain tumors. However, most of them focused on assessing the relationship between the folate intake of pregnant women and the risk of brain tumors in their offspring. Milne et al. collected 327 brain tumor cases and 867 healthy controls based on 10 Australian pediatric oncology centers and collected maternal folate intake through a questionnaire and found that higher folate intake during pregnancy significantly reduced the risk of brain tumors in offspring (OR = 0.60, 95%CI: 0.38–0.98), and this association was also found in low-grade gliomas (OR = 0.44, 95%CI: 0.22–0.89) (33). Another study based on this population also reported that folate in childhood (3–14 years old) was associated with an overall reduced risk of brain tumors (OR = 0.63, 95%CI: 0.41–0.97), especially in low-grade gliomas (OR = 0.52, 95%CI: 0.29–0.92) (32). The study on folate intake and glioma in adults was still insufficient. Our study found the epidemiological evidence for the protective effect of folate intake against gliomas in adults (OR = 0.31, 95%CI: 0.23–0.42), and the results for low-grade gliomas, including astrocytomas, were similar to previous studies, that was, folate intake had a more significant protective effect against low-grade gliomas. The study of serum folate concentration and glioma also found that the proportion of patients with glioma who were lower than the serum folate biological reference value was higher (*p* < 0.001) (63). However, Chen et al. conducted a population-based case–control study in eastern Nebraska and found that dietary folate intake was not associated with the risk of glioma (OR = 0.90, 95%CI: 0.50–1.50) and was considered to be related to the small sample size of the study (64). From the results of the dose–response relationship, the range of folate intake was also consistent with previous studies. It was generally believed that there was a U-shaped relationship between folate and cancer and that when folate intake was between 150 μg/day and 1,000 μg/day, the risk of cancer was significantly reduced (61). Folate is essential for normal DNA synthesis and repair. Its deficiency can activate proto-oncogenes by reducing intracellular S-adenosyl methionine and altering cytosine methylation in DNA, which can also lead to an imbalance of DNA precursors, misincorporation of uracil into DNA and chromosome breakage, which may be the potential mechanism of folate in preventing gliomas (65). In addition, folate can also be used as an adjuvant to assist in treating gliomas. It limited the proliferation of glioma cells and increased the sensitivity to temozolomide-induced apoptosis by inducing DNA methylation (66).

The study of biotin and cancer has mainly focused on biotin as a ligand in the treatment of new drugs (67, 68). Few studies have reported the effect of biotin on cancer risk. Although our study found the protective effect of biotin against glioma, the results were inconsistent in sensitivity analysis, such as different BMI. In different pathological types, biotin was only significantly associated with astrocytoma (OR = 0.55, 95%CI: 0.36–0.84) but not in glioblastoma (OR = 0.81, 95%CI: 0.65–1.01). In different pathological grades, the results of low-grade gliomas and high-grade gliomas were also the opposite. This had to make us carefully consider the relationship between biotin and glioma.

This study had several advantages. First, we explored the relationship between five dietary B vitamins and glioma. As previous studies have paid more attention to the relationship between vitamin A, vitamin C, vitamin E, and glioma, the results of this study supplement the evidence of the preventive effect of B vitamins against glioma. Secondly, the studies on B vitamins and glioma often stayed in cell and animal experiments. The results of this study were mutually confirmed with the existing experimental evidence, especially for thiamine, nicotinic acid, and folate, which were lack of clinical study. Third, we thoroughly explored the relationship between gliomas with different pathological subtypes, pathological grades, and B vitamins. The dose–response relationships between B vitamins and the risk of glioma were described for the first time, which were consistent with the existing dose–response relationship between B vitamins and cancer. These provided further population evidence for the prevention and treatment of glioma by B vitamins. However, there were still some limitations in this study. On the one hand, B vitamins participated in one-carbon metabolism in different forms, such as the synthesis of nucleotides and the metabolism of amino acids, which was of great significance for maintaining normal cell growth. However, because the food composition table failed to provide a broader content of B vitamins, such as vitamin B12, this study only evaluated the five common B vitamins, and could not explore the association between more B vitamins and glioma, especially the interaction between B vitamins. But this has been far the most comprehensive result about B vitamins and gliomas. On the other hand, the association between the concentration of B vitamins in the body and glioma has not been evaluated. Although the food frequency questionnaire had been verified and had good repeatability and validity. However, due to the limitation of methods, there was still a certain bias in the assessment of the intake of B vitamins through the food frequency questionnaire. Assessing the concentration of B vitamins in the body can reduce this uncertainty. Since the concentration of B vitamins in the subjects was not detected in this study, the association between internal exposure and glioma should be evaluated in future studies in combination with the circulating concentration of B vitamins in or biomarkers. In addition, since this study was a case–control study, their causality cannot be verified.

## Conclusion

In summary, in this study of B vitamins and gliomas, we observe that thiamine, riboflavin, nicotinic acid, and folate were associated with a significantly decreased risk of gliomas. Still, the roles of biotin are different in different populations. The relationship between them should be further verified by prospective cohort studies in the future.

## Data availability statement

The original contributions presented in the study are included in the article/Supplementary material, further inquiries can be directed to the corresponding author.

## Ethics statement

The studies involving human participants were reviewed and approved by the Institutional Review Board of Beijing Tiantan Hospital, Capital Medical University (no. KY2022-203-02). The patients/participants provided their written informed consent to participate in this study.

## Author contributions

WL and WZ contributed to the conception or design of the work and wrote the manuscript. WZ, JJ, and SH contributed to data collection and analysis. XK, CW, FC, BZ, and SL contributed to data collection and management. All authors contributed to the article and approved the submitted version.

## Funding

This study was supported by the Talent Introduction Foundation of Tiantan Hospital (no. RCYJ-2020-2025-LWB) and Advanced Research and Training Program of Beijing Double Leading Scholars from China academy of Chinese Medical Science (no. 2-759-02-DR).

## Conflict of interest

The authors declare that the research was conducted in the absence of any commercial or financial relationships that could be construed as a potential conflict of interest.

## Publisher’s note

All claims expressed in this article are solely those of the authors and do not necessarily represent those of their affiliated organizations, or those of the publisher, the editors and the reviewers. Any product that may be evaluated in this article, or claim that may be made by its manufacturer, is not guaranteed or endorsed by the publisher.

## Supplementary material

The Supplementary material for this article can be found online at: https://www.frontiersin.org/articles/10.3389/fnut.2023.1122540/full#supplementary-material
