# Supplementary material for: Dietary B vitamins and glioma: A case–control study based on Chinese population
Source: Front Nutr. 2023 Mar 2;10:1122540. doi: 10.3389/fnut.2023.1122540 (PMC10018137; doi:10.3389/fnut.2023.1122540)
Supplement: Supplementary file 1 [file Data_Sheet_1.docx]

Supplementary Material

Table S1. Correlations between dietary B vitamins among the case-control participants.

Table S2. Sensitivity analysis of B vitamins and glioma.

Table S1. Correlations between dietary B vitamins among the case-control participants.

|  | Thiamine | Riboflavin | Nicotinic acid | Folate | Biotin |
| --- | --- | --- | --- | --- | --- |
| Thiamine | 1.000 | 0.761 | 0.798 | 0.710 | 0.642 |
| Riboflavin |  | 1.000 | 0.714 | 0.774 | 0.687 |
| Nicotinic acid |  |  | 1.000 | 0.581 | 0.559 |
| Folate |  |  |  | 1.000 | 0.744 |
| Biotin |  |  |  |  | 1.000 |

* *P values* of all correlation coefficients in the table were less than 0.01.

Table S2. Sensitivity analysis of B vitamins and glioma.

| Group ^a^ | Model 1^b^ | *P-value* | Model 2^c^ | *P-value* |
| --- | --- | --- | --- | --- |
| **Age** |  |  |  |  |
| **≤40(n=500)** |  |  |  |  |
| Thiamine | 0.98（0.94-1.02） | 0.242 | 0.89(0.84-0.94) | ＜0.001 |
| Riboflavin | 0.94（0.90-0.98） | 0.002 | 0.73(0.67-0.80) | ＜0.001 |
| Nicotinic acid | 0.94（0.84-1.06） | 0.310 | 0.52(0.41-0.67) | ＜0.001 |
| Folate | 0.72（0.63-0.83） | ＜0.001 | 0.40(0.31-0.52) | ＜0.001 |
| Biotin | 1.04（0.97-1.12） | 0.280 | 0.84(0.73-0.97) | 0.019 |
| **＞41(n=512)** |  |  |  |  |
| Thiamine | 0.91（0.87-0.95） | ＜0.001 | 0.84(0.77-0.90) | ＜0.001 |
| Riboflavin | 0.90（0.87-0.94） | ＜0.001 | 0.74(0.68-0.81) | ＜0.001 |
| Nicotinic acid | 0.81（0.72-0.92） | 0.001 | 0.62(0.49-0.77) | ＜0.001 |
| Folate | 0.56（0.48-0.65） | ＜0.001 | 0.29(0.22-0.39) | ＜0.001 |
| Biotin | 0.88（0.82-0.96） | 0.002 | 0.76(0.66-0.87) | ＜0.001 |
| **Sex** |  |  |  |  |
| **Male(n=568)** |  |  |  |  |
| Thiamine | 0.98（0.95-1.01） | 0.118 | 0.93(0.89-0.97) | ＜0.001 |
| Riboflavin | 0.94（0.91-0.97） | 0.001 | 0.73(0.67-0.79) | ＜0.001 |
| Nicotinic acid | 0.96（0.87-1.06） | 0.445 | 0.70(0.59-0.84) | ＜0.001 |
| Folate | 0.68（0.60-0.77） | ＜0.001 | 0.37(0.29-0.46) | ＜0.001 |
| Biotin | 0.97（0.91-1.04） | 0.361 | 0.79(0.70-0.89) | ＜0.001 |
| **Female(n=444)** |  |  |  |  |
| Thiamine | 0.85（0.80-0.90） | ＜0.001 | 0.67(0.58-0.79) | ＜0.001 |
| Riboflavin | 0.89（0.85-0.94） | ＜0.001 | 0.78(0.70-0.86) | ＜0.001 |
| Nicotinic acid | 0.67（0.57-0.80） | ＜0.001 | 0.37(0.25-0.54) | ＜0.001 |
| Folate | 0.58（0.49-0.68） | ＜0.001 | 0.35(0.26-0.48) | ＜0.001 |
| Biotin | 0.96（0.88-1.05） | 0.370 | 0.81(0.66-0.98) | 0.027 |
| **BMI** |  |  |  |  |
| **≤23.31(n=506)** |  |  |  |  |
| Thiamine | 0.92（0.88-0.97） | 0.001 | 0.85(0.78-0.92) | ＜0.001 |
| Riboflavin | 0.91（0.87-0.95） | ＜0.001 | 0.79(0.72-0.87) | ＜0.001 |
| Nicotinic acid | 0.82（0.72-0.94） | 0.005 | 0.58(0.44-0.75) | ＜0.001 |
| Folate | 0.67（0.58-0.77） | ＜0.001 | 0.45(0.35-0.59) | ＜0.001 |
| Biotin | 0.97（0.89-1.05） | 0.389 | 0.88(0.75-1.02) | 0.093 |
| **＞23.31(n=506)** |  |  |  |  |
| Thiamine | 0.95（0.92-0.99） | 0.008 | 0.91(0.86-0.96) | ＜0.001 |
| Riboflavin | 0.93（0.89-0.96） | ＜0.001 | 0.73(0.67-0.79) | ＜0.001 |
| Nicotinic acid | 0.88（0.79-0.99） | 0.029 | 0.59(0.48-0.73) | ＜0.001 |
| Folate | 0.60（0.52-0.69） | ＜0.001 | 0.31(0.24-0.41) | ＜0.001 |
| Biotin | 0.95（0.88-1.02） | 0.148 | 0.76(0.67-0.87) | ＜0.001 |
| **Education level** |  |  |  |  |
| **University and above(n=627)** |  |  |  |  |
| Thiamine | 0.95（0.92-0.99） | 0.010 | 0.85(0.79-0.91) | ＜0.001 |
| Riboflavin | 0.94（0.91-0.97） | 0.001 | 0.76(0.70-0.82) | ＜0.001 |
| Nicotinic acid | 0.92（0.82-1.02） | 0.122 | 0.60(0.49-0.74) | ＜0.001 |
| Folate | 0.66（0.58-0.75） | ＜0.001 | 0.38(0.30-0.47) | ＜0.001 |
| Biotin | 0.98（0.91-1.05） | 0.554 | 0.79(0.69-0.91) | 0.001 |
| **Household income** |  |  |  |  |
| **>3,000 ¥/month(n=871)** |  |  |  |  |
| Thiamine | 0.94（0.92-0.97） | ＜0.001 | 0.90(0.86-0.94) | ＜0.001 |
| Riboflavin | 0.91（0.88-0.94） | ＜0.001 | 0.75(0.71-0.80) | ＜0.001 |
| Nicotinic acid | 0.84（0.77-0.92） | ＜0.001 | 0.63(0.54-0.74) | ＜0.001 |
| Folate | 0.61（0.54-0.68） | ＜0.001 | 0.38(0.31-0.46) | ＜0.001 |
| Biotin | 0.94（0.89-0.99） | 0.041 | 0.79(0.71-0.88) | ＜0.001 |
| **Smoking status** |  |  |  |  |
| **Never smoking(n=735)** |  |  |  |  |
| Thiamine | 0.92（0.88-0.95） | ＜0.001 | 0.83(0.76-0.89) | ＜0.001 |
| Riboflavin | 0.92（0.88-0.95） | ＜0.001 | 0.79(0.74-0.85) | ＜0.001 |
| Nicotinic acid | 0.78（0.70-0.88） | ＜0.001 | 0.51(0.41-0.63) | ＜0.001 |
| Folate | 0.65（0.58-0.73） | ＜0.001 | 0.43(0.35-0.52) | ＜0.001 |
| Biotin | 0.95（0.89-1.02） | 0.149 | 0.79(0.70-0.89) | ＜0.001 |
| **History of allergies** |  |  |  |  |
| **No(n=899)** |  |  |  |  |
| Thiamine | 0.94（0.92-0.97） | ＜0.001 | 0.89(0.85-0.94) | ＜0.001 |
| Riboflavin | 0.91（0.88-0.94） | ＜0.001 | 0.74(0.69-0.79) | ＜0.001 |
| Nicotinic acid | 0.85（0.78-0.93） | 0.001 | 0.59(0.50-0.70) | ＜0.001 |
| Folate | 0.63（0.56-0.70） | ＜0.001 | 0.38(0.32-0.46) | ＜0.001 |
| Biotin | 0.96（0.91-1.02） | 0.202 | 0.83(0.75-0.92) | ＜0.001 |
| **Family history of cancer** |  |  |  |  |
| **No(n=753)** |  |  |  |  |
| Thiamine | 0.95（0.91-0.98） | 0.001 | 0.89(0.85-0.94) | ＜0.001 |
| Riboflavin | 0.92（0.88-0.95） | ＜0.001 | 0.75(0.70-0.81) | ＜0.001 |
| Nicotinic acid | 0.89（0.80-0.98） | 0.021 | 0.63(0.53-0.76) | ＜0.001 |
| Folate | 0.60（0.53-0.68） | ＜0.001 | 0.36(0.29-0.44) | ＜0.001 |
| Biotin | 0.94（0.88-1.01） | 0.071 | 0.78(0.69-0.88) | ＜0.001 |

a. Unconditional logistic regression model was used for sensitivity analysis.

b. Model 1: Unadjusted model.

c. Model 2: adjusted covariates in model 2 (except for corresponding hierarchical variables).
